# Transitioning From Preclinical Evidence to Advanced Therapy Medicinal Product: A Spanish Experience

**DOI:** 10.3389/fcvm.2021.604434

**Published:** 2021-02-04

**Authors:** Paloma Gastelurrutia, Cristina Prat-Vidal, Joaquim Vives, Ruth Coll, Antoni Bayes-Genis, Carolina Gálvez-Montón

**Affiliations:** ^1^Institut d'Investigació Biomèdica de Bellvitge-IDIBELL, L'Hospitalet de Llobregat, Spain; ^2^Insuficiencia Cardíaca y Regeneración Cardíaca Research Program, Fundació Institut d'Investigació en Ciències de la Salut Germans Trias i Pujol (IGTP), Badalona, Spain; ^3^Centro de Investigación Biomédica en Red Cardiovascular, Instituto de Salud Carlos III, Madrid, Spain; ^4^Servei de Teràpia Cel·lular, Banc de Sang i Teixits, Barcelona, Spain; ^5^Musculoskeletal Tissue Engineering Group, Vall d'Hebron Research Institute (VHIR), Universitat Autònoma de Barcelona, Barcelona, Spain; ^6^Departament de Medicina, Universitat Autònoma de Barcelona, Barcelona, Spain; ^7^Hospital Universitari Germans Trias i Pujol, Badalona, Spain; ^8^Department of Medicine, Universitat Autònoma de Barcelona, Barcelona, Spain

**Keywords:** advanced therapy medicinal product, investigational medicinal product, investigational medicinal product dossier (IMPD), translational (regulation), regulatory agency

## Abstract

A systematic and ordered product development program, in compliance with current quality and regulatory standards, increases the likelihood of yielding a successful advanced therapy medicinal product (ATMP) for clinical use as safe and effective therapy. As this is a novel field, little accurate information is available regarding the steps to be followed, and the information to be produced to support the development and use of an ATMP. Notably, successful clinical translation can be somewhat cumbersome for academic researchers. In this article, we have provided a summary of the available information, supported by our experience in Spain throughout the development of an ATMP for myocardial infarction, from the pre-clinical stage to phase I clinical trial approval.

## Introduction

Recent years have brought tremendous developments in cardiac tissue engineering (1). Cell therapy approaches have been attempted for the repair of scars left by myocardial infarction (MI); however, clinical trials have reported only modest and clinically irrelevant improvements in cardiac function (2–4). Thus, the research focus has shifted to cardiac tissue engineering approaches that can deliver cells to the injured myocardium with supportive scaffolds, thus improving cell retention and survival (5). Successful clinical translation of these engineered constructs requires that specific steps be followed from scientific, quality, and regulatory standpoints. First, they have to be conceived and developed in a basic research laboratory. Second, they need to be confirmed as safe and effective in relevant animal models. Third, they must be adapted to current good manufacturing practice (GMP)-compliant procedures for obtaining an advanced therapy medicinal product (ATMP). Finally, the ATMP has to be tested in patients in the context of a clinical trial.

## Sections on Assessment of Policy/Guidelines Options and Implications

In 2003, the concept of ATMP was defined and introduced in European legislation. ATMP originally comprised gene therapy medicinal products (GTMPs) and somatic cell therapy medicinal products (CTMPs), with the later inclusion of tissue-engineered products (TEPs) in Regulation (EC) No. 1394/2007 (6, 7). The ATMP industry is still emerging, and many therapy proposals in this field are led by independent researchers who have little, if any, process development experience (8).

ATMPs are ground-breaking medicines based on the use of genes, tissues, or cells for the treatment of human disease and injury. They are classified into four main types: GTMPs, CTMPs, TEPs, and combined ATMPs. GTMPs contain genes that have therapeutic, prophylactic, or diagnostic effects. CTMPs include cells or tissues that have been substantially manipulated to change their biological characteristics, physiological functions, or structural properties, or that are intented to be used for a purpose other than their essential functions in the body, with the aim of treating, preventing, or diagnosing a disease. TEPs contain modified cells or tissues of human and/or animal origin (viable or non-viable) that are used to repair, regenerate, or replace human tissue (9). Finally, combined ATMPs contain a medical device as an integral part of the product (10). They may also contain additional substances, such as biomaterials, chemicals, scaffolds, or matrices. The mechanisms of action of ATMPs include pharmacological, immunological, or metabolic activities (10).

Due to the novelty and complexity of ATMPs, there is a lack of accurate information regarding the steps required for their clinical translation, and data that must be produced to achieve regulatory agency approval. This can make it difficult for independent researchers from an academic environment to successfully get an ATMP to the patient population. Investigational medicinal product (IMP) development can be challenging in many aspects, including materializing a research idea, cell/tissue isolation and characterization, preclinical studies to prove safety and efficacy, scaling-up prototypes for human use, defining manufacturing processes in accordance with current GMP, and meeting all ethics and regulatory requirements (10–14). Our group recently developed a pioneering ATMP, named PeriCord, that is presently being tested in humans for cardiac repair after MI. PeriCord comprises a human decellularized pericardial matrix enriched with Wharton's jelly-derived mesenchymal stromal cells (15). Here, we report our experience in creating this IMP in Spain, and through its translation from preclinical stages to use in patients (15).

### Four Stages Along the Journey

#### Preclinical Stage

Basic research laboratories can generate regenerative/repair-driven hypotheses and test them in animal models. To achieve clinical translation, a newly developed ATMP must be proven safe and effective in a planned preclinical program including studies addressing pharmacodynamics (PD), pharmacokinetic (PK), and toxicology (Tox) (16, 17). For a product to be proven safe, the risk-benefit profile must be assessed as acceptable for the target disease or condition, the product must be well-characterized to reduce batch variability and ensure the expected function, and the genetic stability must be confirmed. Accurate characterization of the product is also important for avoiding differences in effectivity, ensuring consistency from dose to dose (11). Thus, as soon as possible, it is important to define the identity, viability, purity, and potency of the ATMP.

Experiments in animal models should be conducted to evaluate the biodistribution of an ATMP in principal organs (e.g., brain, liver, spleen, lungs, kidneys, and gonads), to identify target organs and tissues (18), and to assess local effects. Studies must review and confirm the expected mechanism of action of the product's active ingredient, i.e., the biologically active ingredient that exerts the expected therapeutic effect. Animal experiments should also reveal an absence of tumorigenic activity and an acceptable safety profile. In terms of effectiveness, animal models may prove efficacy by confirming the hypothesis at the preclinical level. In some cases, no animal model is available for the specific pathology under investigation. In this case, a new model can be designed if the basic research group has sufficient experience in the pre-clinical large animal setting, and can fine-tune a model to mimic a human pathological scenario. However, sometimes a disease cannot be reproduced due to its high complexity or mortality. In this scenario, pre-clinical studies can be performed using different animal models that present the different symptoms separately.

Prior to administration to a patient, the product must comply with the above-described requirements, as well as others.

#### Translational Stage

We refer as the translational stage, the phase during which the researchers have a proof of concept and are ready to start working to develop a product for clinical use. Several considerations must be taken into account for products intended for human use:

1. The preclinical product must be adapted to fulfill the regulatory and legal requirements for human use. To this end, the ATMP must be manufactured following procedures in compliance with current GMP standards and in GMP-accredited facilities. If the preclinical product was not originally produced using GMP procedures and/or clinical grade reagents, it should be modified. Due to the potential impact on the final product's safety and quality, ATMP developers must make available the quality attributes and composition of all raw materials used in the manufacturing process. Importantly, this information is required for the regulatory submission, especially when animal-derived components are used. Any raw materials and excipients must be suitable for clinical use, and some may have to be substituted for others with similar characteristics upon appropriate risk analysis and experimental testing (e.g., xeno-free, clinical grade ingredients) (19), which may be more expensive. Additionally, sometimes extensive *ex vivo* expansion is required to yield sufficient cell numbers for clinical use, and scale-up processes should be defined and incorporated within the manufacturing procedure to adapt doses and/or final product dimensions to human scale (20).
2. For autologous cell products, “scaling-out” usually requires the ability to conduct multiple parallel bioprocesses by using traditional planar 2D cultures for the production of larger amounts of finished product (20, 21). For unmatched allogeneic products, “scaling-up” involves the use of expansion platforms, such as bioreactors, to entail reproducible and robust manufacturing bioprocess, therefore offering major advantages with respect to GMP implementation.

Some specific documents must be generated to collect all of the information required by the competent authorities for their evaluation and approval of the IMP, and their approval of a clinical trial together with the local ethics committee. These aspects will be detailed in the section ATMP approval for clinical trials.

#### Production Stage

Several issues must be addressed prior to the actual production of an ATMP.

1. GMP compliance: The ATMP manufacturing facilities must comply with current GMP, thus guaranteeing the quality and safety of the manufactured products. This accreditation is granted by the competent authorities, e.g., the AEMPS in Spain. GMP is a quality management system to ensure that products are consistently processed and controlled according to quality standards. This system guarantees traceability at all times, covering every aspect of manufacturing and distribution. GMP covers all aspects of production, from the starting materials, premises, and equipment to staff training and personal hygiene. It is essential to have detailed written procedures (standard operating procedures; SOP) for each process that could affect the finished product quality, which are followed each time a product is manufactured (22). Quality control documents are required to register all utilized processes and materials, including batch records and expiration dates. The products commonly used in preclinical studies are often unsuitable for clinical use. For some such ingredients, excipients, or culture reagents, there is an equivalent, generally more expensive, GMP-compliant alternative. However, other materials may be more difficult to replace. For example, bovine serum has been widely used in cell culture for over a century (23), but the current trend is to switch from the use of serum or platelet lysate to serum-free chemically defined media formulations to alleviate concerns regarding safety (i.e., xenoviral transfer and contamination), efficacy (batch-to-batch variation), and scarcity (11, 20, 23).
2. Reproducibility, safety, and consistency: There are several critical process stages for assuring consistency and meeting the established specifications of the finished drug product (DP)—including tissue harvesting, cell isolation and expansion, cell and finished product storage, lot size (number of cells per production batch), and methods. The manufacturing bioprocess is designed to minimize the risks of deviating from specifications along the production process, such that there is no need to solely rely on testing of the finished product. This is known as “quality by design (QbD),” an approach that aims to ensure product quality by combining statistical, analytical, and risk-management methodologies into the design, development, and manufacturing processes (24– 26). Acceptance and release criteria must be determined to ensure the safety and consistency of the final DP, with regards to microbiological growth (negative serological and microbiological testing), physiochemical properties, dose range, cell viability, and endotoxin levels, among other concerns.
3. Scalability: Process scalability is a major factor to consider when starting from a preclinical product and aiming to develop its corresponding IMP format. For translational allogeneic cell therapy that requires cells, reagents, and culture media, large numbers of cells are needed to achieve a certain dose ready for use in several patients, thus affecting product cost and quality (17). The cells must retain their key quality attributes of identity, potency, purity, and safety, and there must be no changes in quality with regards to cell differentiation capability (11). Quality by design strategies and classical experimental design approaches can be applied to optimize cell culture media, reagents, and process parameters to create a robust, reproducible, and scalable process (11, 27).
4. Stability: The DP stability period must be determined based on cell stability and the product life cycle (28). Cells can multiply, but do not have to multiply continuously, only when needed for their normal cycle. When they multiply, they must maintain a stable karyotype and stable expression of cytokine markers and telomerase. The product life cycle is the process a product goes through once it is released until it is removed upon reaching its expiration date. This shelf-life is determined based on changes in the product characteristics, and is assessed in the preclinical stage and during product development, and might be reassessed during the clinical trial phase according to the product stability protocols.

All of these factors must be considered to manufacture a clinically appropriate product adapted from the preclinical version. The end results must be a comparable product in terms of identity, purity, potency, and safety (29).

#### ATMP Approval for Clinical Trials

**Figure 1:**
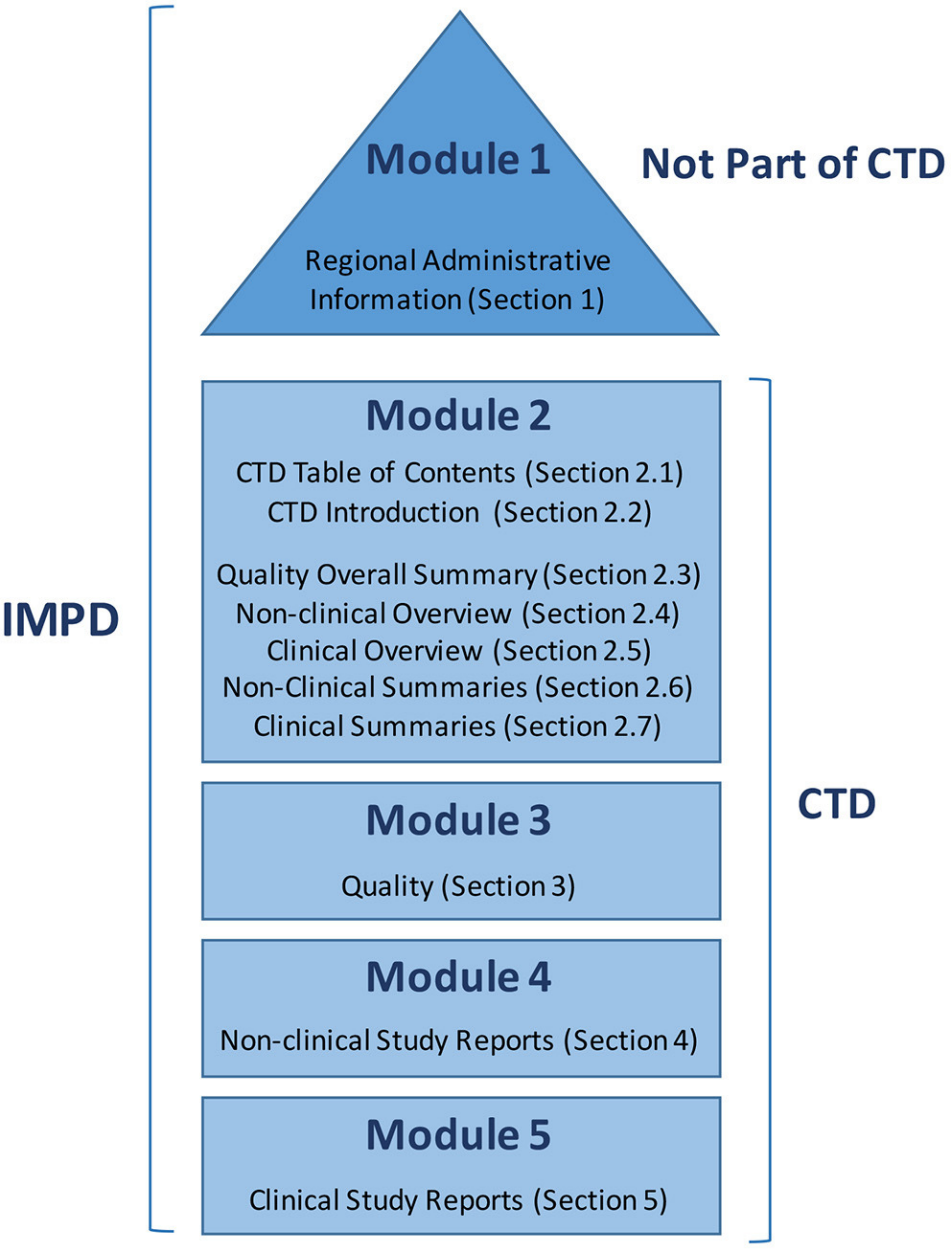
Parts of the Investigational Medicinal Product Dossier (IMPD) as collected in the Notice to Applicants prepared by the European Commission.

After the preclinical and translational stages, and upon achieving ATMP manufacturing and validation, all required information must be presented to the competent authority and final approval obtained to commence a clinical trial. Here we summarize all of the documentation required for presentation to the Spanish Agency of Medicines and Medical Devices (AEMPS):

1. Target Product Profile (TPP): This is not a mandatory document in Spain, but rather a summary of the necessary information about the product development process. A TPP is created with the aim of ensuring that the drug development process is efficient and provides all of the required medical, technical, and scientific information about a product (30). It can be worthwhile to start the TPP at an early stage (preclinical stage) to provide a clear vision of the whole process.
2. Investigational Medicinal Product Dossier (IMPD): The IMPD, including modules 1 to 5 (described below), is one of the main IMP-related documents required for clinical trial approval in one or more European Union Member States. The IMPD includes regional administrative information and summaries of the data related to the quality, manufacture, and control of any IMP (including both reference product and placebo), as well as data from non-clinical and clinical studies (Figure 1).

The AEMPS does not offer guidance on writing an IMPD, but suggests following the European Guidance from the European Medicines Agency (EMA) (31). Figure 1 shows the parts of the IMPD as summarized in a Notice to Applicants (32) prepared by the European Commission in consultation with competent authorities of the member states, the EMA, and other interested parties as a guideline for fulfilling the obligations raised by Article 6 of Regulation (EC) No. 726/2004, and with respect to Annex I of Directive 2001/83/EC (European Commission Notice to Applicants). Based on this Notice to Applicants, we adapted the index for our IMP, as shown in Table 1.

**Table 1:** Investigational medicinal product dossier (IMPD) index adapted from the Notice to applicants by the European Commission.

**General index**	**Module 2**	**Module 3**	**Module 4**
Module 2Module 3Module 4	2.1. Index 2.2. Introduction 2.3. Quality Overall Summary 2.4. Non-clinical Overview 2.5. Clinical Overview 2.6. Written and Tabulated Non-clinical Summaries 2.6.1 Introduction 2.6.2 Pharmacology 2.6.3 Pharmacology overview 2.6.4 Pharmacokinetics 2.6.5 Toxicology2.7. Written and Tabulated Clinical Summaries 2.7.1 Biopharmaceutical studies and related analytical methods 2.7.2 Clinical pharmacology studies 2.7.3 Clinical efficacy studies 2.7.4 Clinical safety studies 2.8. References 2.9. Studies Synopsis	3.1. Index 3.2. Body of Data 3.2.S Active ingredient (AI) 3.2.S.1 General information 3.2.S.2 Production 3.2.S.3 AI characterization 3.2.S.4 Specifications 3.2.S.5 Packaging description 3.2.S.6. Stability 3.2.P Final product (FP) 3.2.P.1 Product description and composition 3.2.P.2 Pharmaceutical development 3.2.P.3 Production 3.2.P.4 Specifications 3.2.P.5 FP control 3.2.P.6 Packaging 3.2.P.7 Stability 3.2.P.8 FP batch analysis 3.3. References	4.1. Index 4.2. Studies Reports 4.3. MSCs Toxicity and Biodistribution Studies 4.3.1. Intrathecal and systemic administration of MSCs, WJ in an immunodeficient murine model of traumatic spinal cord injury 4.3.2. Administration of MSCs carried through fibrin matrix in an immunodeficient murine myocardial infarction model 4.4. Multipotency oF MSC,WJ 4.5. Immunomodulation Studies 4.6. Genetic Stability of MSC,WJ 4.7. *In vivo* Efficacy Studies 4.7.1. Efficacy study of the implantation of MSCs carried by matrices in the murine myocardial infarction model 4.7.2. Efficacy study of the implantation of MSCs carried by matrices in the porcine myocardial infarction model 4.8. References

The IMPD includes the following modules:

- Module 1: This section is not a part of the Common Technical Document (CTD), and includes all regional administrative information required by the AEMPS.
- Module 2: This module provides an overview of all of the information, divided into several sections, including a table of contents (2.1), introduction (2.2), review of the quality information (2.3), review of the non-clinical data (2.4), review of the clinical data (2.5), and two tabulated summaries: one for the non-clinical data (2.6) and one for the clinical data (2.7).
- Module 3: This section is a compendium of the available quality information, which will also be sent as a discrete document in Block I of the required documentation for the Ethics Committee and AEMPS evaluation (see below). The document includes data regarding the characterization, fabrication, specifications, stability, packaging, and quality control analysis of the active ingredient and the finished product (i.e., the ATMP).
- Module 4: This module constitutes a compendium of the available non-clinical data, which includes toxicity and biodistribution studies, mechanism of action, and safety data. For TEPs, this includes information concerning the genetic stability of the active cells, and *in vivo* efficacy studies.
- Module 5: This non-compulsory module includes clinical study reports, when available.

This structure indicates the necessary experiments for gathering all of the required data in terms of quality and fabrication (Module 2, section Quality overall summary, and Module 3), as well as the pharmacological mechanism of action, pharmacokinetics, toxicity, biodistribution, and safety (Module 4). The IMPD serves as an excellent tool for defining all necessary preclinical studies, and for aligning manufacturing process requirements with product specifications (11).

Common Technical Document (CTD): The International Council for Harmonization of Technical Requirements for Pharmaceuticals for Human Use (ICH) has the mission of bringing together regulatory authorities and pharmaceutical industries worldwide to ensure that safe, effective, and high-quality medicines are developed and registered in the most resource-efficient manner (33). Toward this aim, they created a common format, the CTD (Figure 1), to gather information regarding quality, safety, and efficacy. Module 1 is region-specific, while Modules 2, 3, 4, and 5 are intended to be common among all regions—as has been mandatory since July 2003 in the EU and Japan, and is the strongly recommended format of choice in the United States.
Protocol: The protocol describes how the clinical trial will be conducted, and is the most important document for clinical trial execution. The protocol must include a title; a protocol number, version number, and date; the study objective and justification, containing all relevant previous clinical and preclinical data; the study design; candidate inclusion and exclusion criteria; intervention or treatment; risks and benefits, including adverse reaction definition and reporting; expected trial start and end dates; study withdrawal and blind-opening, if applicable; data management, and statistical analysis; ethical and regulatory considerations; document archive and font documents; quality control; funding; publication policy; references; and annexes. It is highly recommended that this document include a figure explaining the study design and a table detailing the scheduled study visits.
Informed consent form: Informed consent is the procedure guaranteeing that a subject gives consent to participate in a research study, having and understanding all of the information related to the trial objectives, its possible benefits, the risks and inconveniences, the available alternatives, and his/her rights and responsibilities. The candidate subject must have time to think and ask any necessary questions. All of the provided information must be collected in a document with a version number updated with current legislation, and two printed copies must be signed, dated, and kept by the investigator and the patient.
Investigator's Brochure (IB): This document is addressed to the researchers involved in the trial, providing a compilation of the clinical and non-clinical data regarding the product, and the reference safety information. The IB includes all of the IMP-related information from the IMPD that the investigator will need to correctly manage the IMP and the included subjects, ensuring their understanding of the study rationale, IMP dose, dose range, administration, and safety monitoring parameters. The IB should include a title page; summary; table of contents; introduction; section describing the product's physical, chemical, and pharmaceutical properties; non-clinical studies (pharmacology pharmacokinetics, and toxicology); effects in humans (pharmacokinetics, safety, and efficacy); and a summary for the investigator. If an event that is not listed in this document occurs during the trial, the investigator must consider it a non-expected adverse event, and must notate it as such. The contents are detailed in the guideline EMA/CHMP/ICH/135/1995 and the required information is obtained from the IMPD (34).

### Competent Authorities Involved

#### National Level: Competent Authority Correspondence, Approval Request, and Clinical Trial Registration

The AEMPS is the national regulatory agency in Spain. There are several different ways of corresponding with the AEMPS. First, for preliminary specific questions related to the proposed study, enquiry e-mails can be sent to the Support Office for Independent Clinical Research, Clinical Trials Division, Pharmaceutical Medicines for Human Use Department.

**Figure 2:**
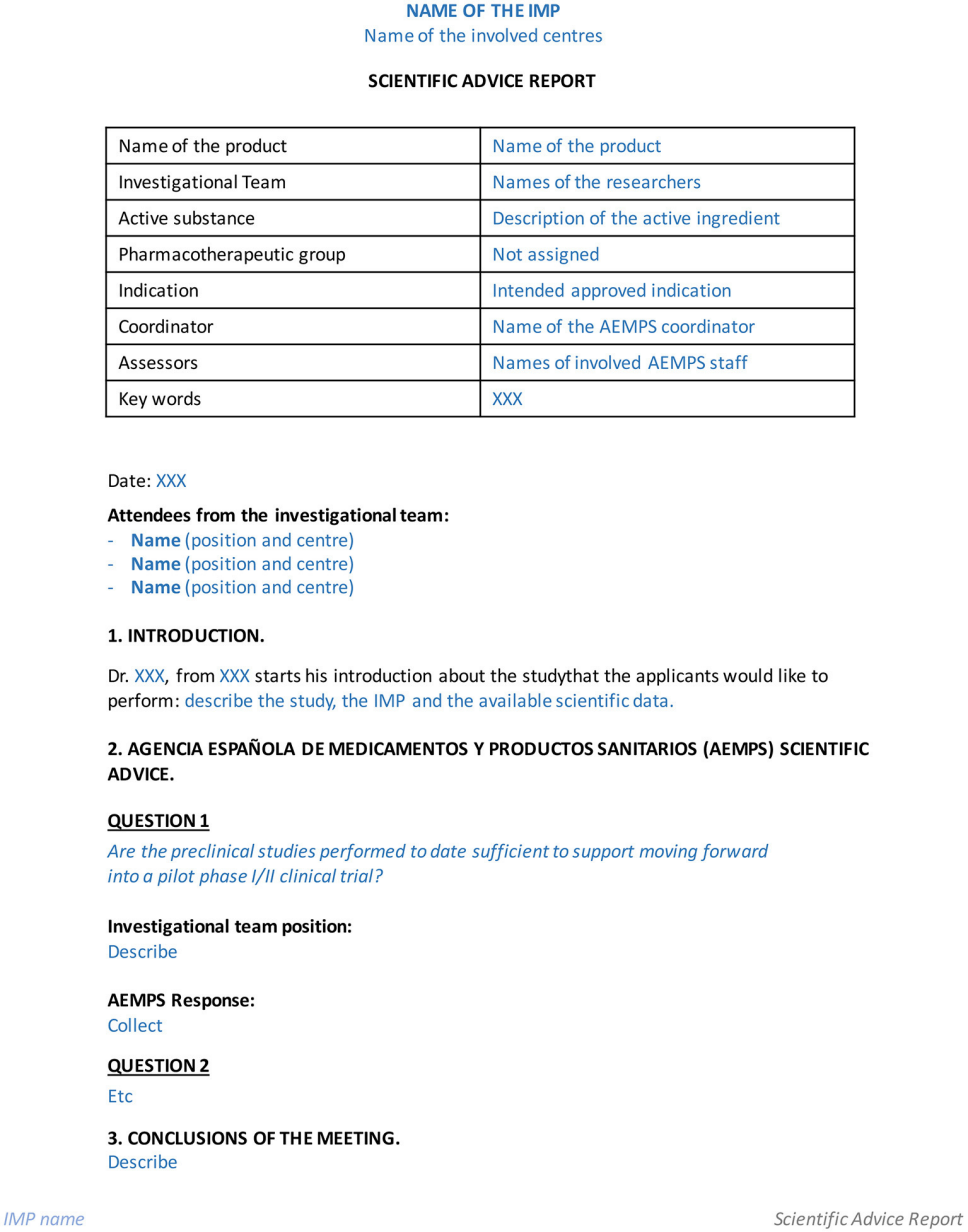
Template to write minutes after obtaining scientific advice from the Spanish Agency of Medicines and Medical Devices (AEMPS).

Second, in the early stages, when considering moving toward the clinical phase, scientific advice can be sought. Scientific advice meetings are face-to-face sessions at AEMPS facilities, at which researchers present their intentions and the questions that they want to discuss with experts from the regulatory agency. These professionals act as assessors for the investigational team, and include representatives from different areas, such as the Division of Biological Products, Advanced Therapies and Biotechnology, and Department of Medical Devices. After the meeting, correspondence occurs between the experts and the Quality Research Department, Pharmacology and Clinical Assessment Division, Medicines for Human Use Department, to whom minutes of the meeting must be sent. Figure 2 presents a template for summarizing meeting minutes. This document is revised and accepted by the AEMPS, and will be part of the collected documentation required to obtain regulatory approval for the clinical trial.

**Figure 3:**
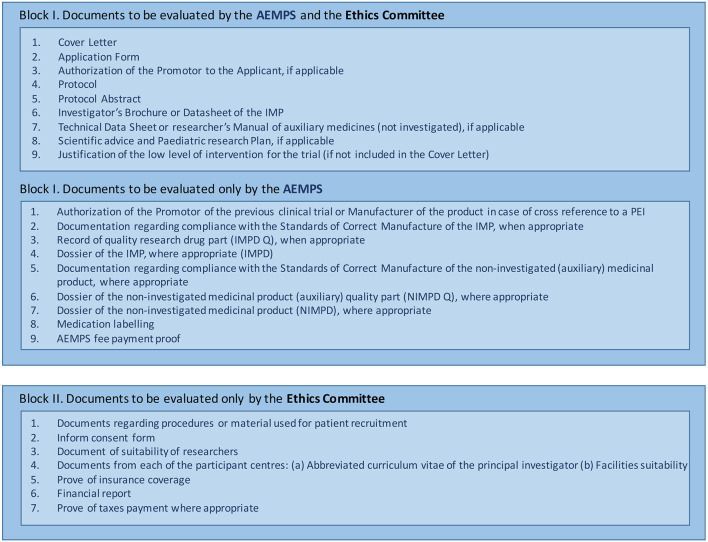
List of documents to upload to the Spanish Agency of Medicines and Medical Devices (AEMPS) platform for investigational medicinal product (IMP) approval.

**Figure 4:**
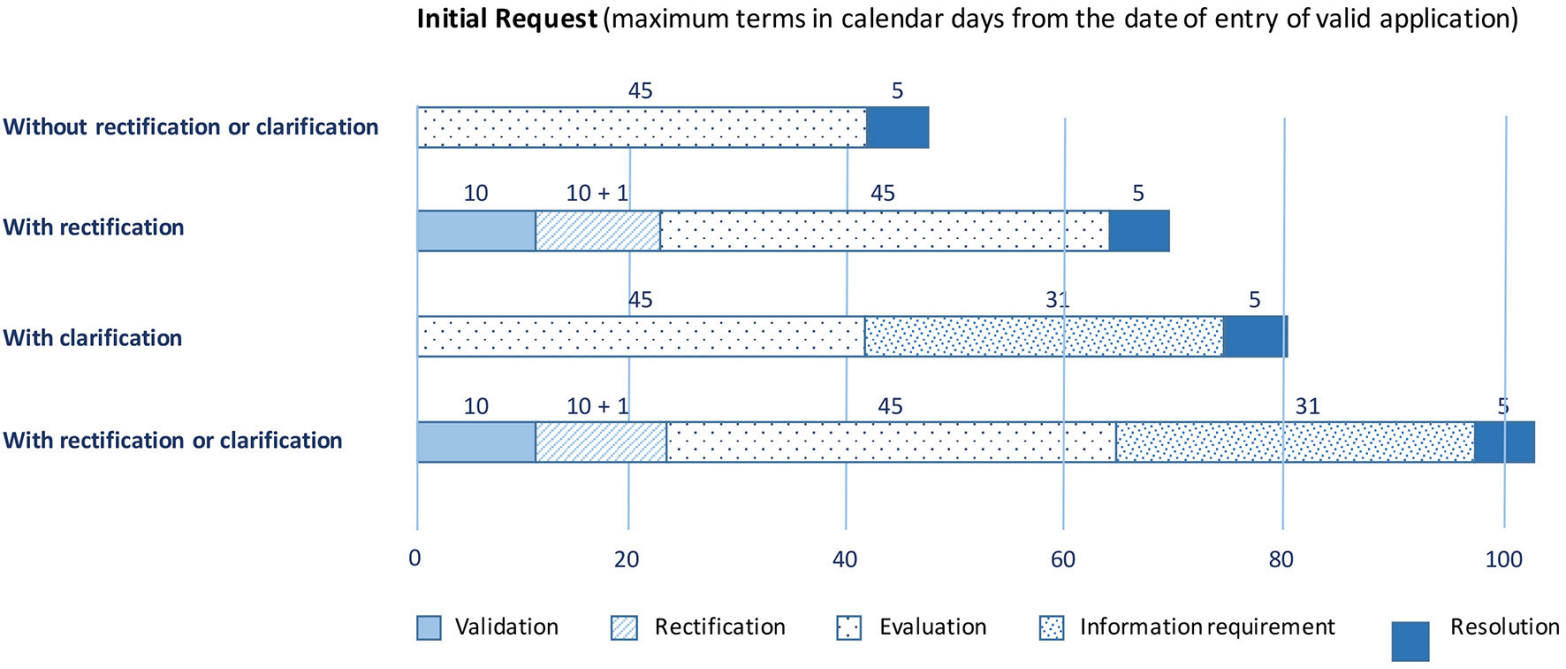
Due dates after Spanish Agency of Medicines and Medical Devices (AEMPS) document submission.

Third, the documentation is submitted via the AEMPS platform (https://ecm.aemps.es/). Figure 3 shows the list of documents that must be uploaded to the platform (35). The evaluation is separated into two main blocks. Block I includes the documents that must be sent to the AEMPS and to the local ethics committee. Block II comprises documents to be sent exclusively for local ethics committee evaluation. The AEMPS must validate Block I, and the local ethics committee must validate Blocks I and II within a maximum of 10 natural days (Figure 4) (36). Both organizations can request rectifications and/or clarifications and the process can be extended over time. Upon approval by the AEMPS and the local ethics committee, the clinical trial must be publicly registered in the Spanish Clinical Trial Registry (REEC, *from Registro Español de Estudios Cl*í*nicos*; https://reec.aemps.es/).

#### European Level: EMA Communication and EU Clinical Trials Registry

The European Regulation on ATMPs has introduced a classification procedure that gives developers the opportunity to verify whether their product can be considered an ATMP. This procedure is optional, free of charge, and may occur at any stage of ATMP development prior to applying for marketing authorization. There is also the possibility of briefing meetings for preparing ATMP classification, which are organized by the EMA Innovation Task Force and represent a starting point for interactions between the European agency and the ATMP developers (6), enabling the EMA to provide scientific advice. However, AEMPS decisions are harmonized with decision by the EMA, and it is not necessary to seek both.

After local regulatory approval, the clinical trial should be registered in the European clinical trials database (EudraCT, https://eudract.ema.europa.eu/eudract-web/index.faces) to allow public consultation of updated study-related information.

## Discussion

Our group is a multidisciplinary team—including basic researchers (biologists, biochemists, and veterinarians) and clinical researchers (physicians and pharmacists). This diversity may not be common in basic research groups, which may need to look outside of their group for a clinical perspective early in the translation process. Notably, despite being a quite large multidisciplinary group, we still had to seek a partner with broad experience in production and product quality. This is why we created a consortium with a center that had the knowledge that we were lacking, had GMP procedures established in their daily activity, and possessed all of the facilities needed for product production.

**Figure 5:**
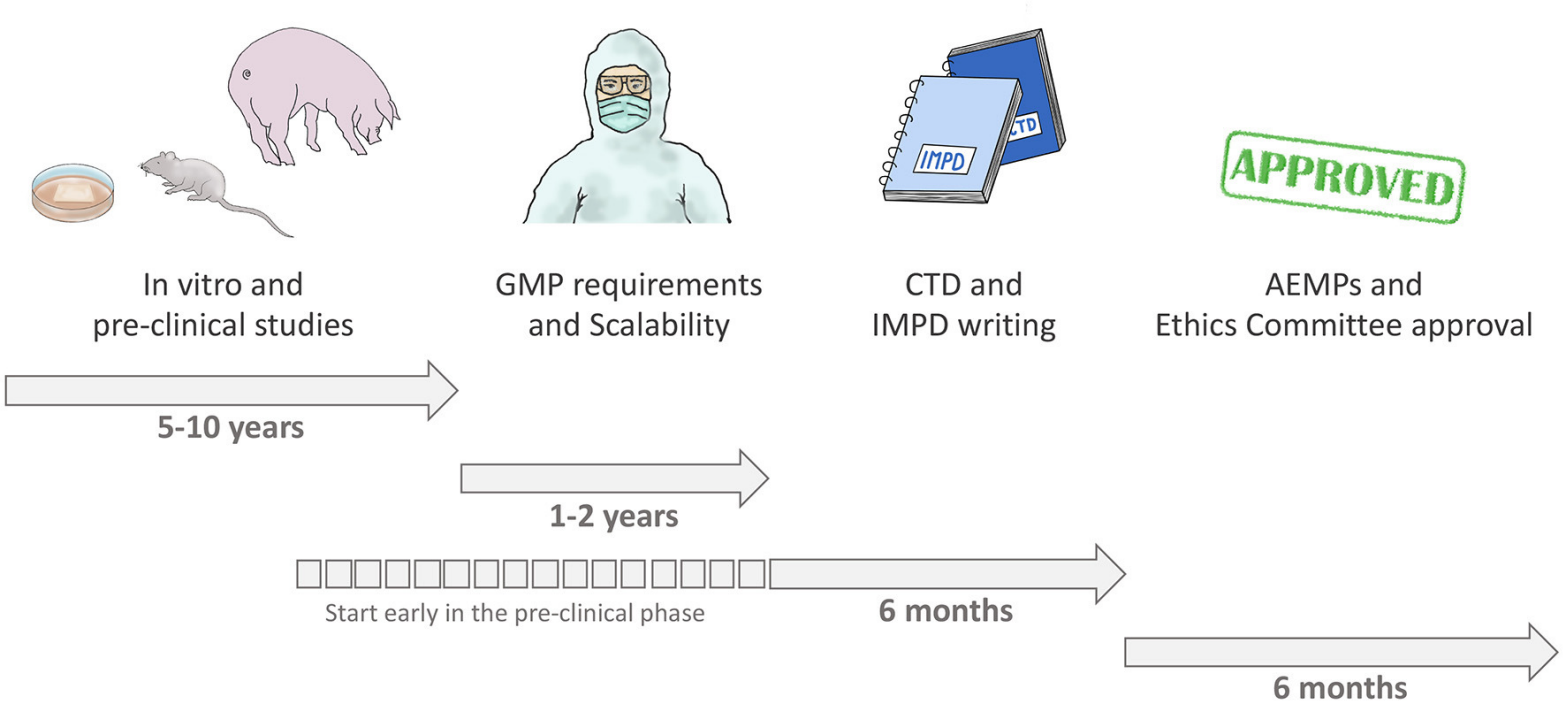
Process of clinical translation.

We had studied cardiac regeneration as a basic research group for almost 10 years. Then we spent over a year to adapt our product and our processes to GMP and, together with our partner center, we generated the IMPD. The optimum approach is to start planning the full process from the very beginning, when a research idea is generated, with the involvement of all experts. Considering the development of an investigational product as a project enables early planning of a scheme that includes preclinical and *in-vivo* animal models and quality aspects, along with bioprocess aspects, as well as compliance with the GMP and legal framework requirements (16). This approach is important to ensure appropriate design of the non-clinical package required for a clinical trial—helping to avoid skipping process steps; setting overly optimistic deadlines; underestimating the financial, time, and personnel requirements; and performing studies without proper documentation (16). Figure 5 depicts the whole process for clinical translation. We performed a lot of work without having considered all GMP requirements, which resulted in a longer overall process. We are now aware of these issues for future projects that we hope to be developing together as a consortium.

In conclusion, basic research laboratories can generate innovative regenerative/repair-driven hypotheses; however, preclinical products must go through a complex process of translational adaptation before reaching patients. Intentions should be discussed with the competent authorities as early as possible, as they may guide researchers to avoid possible errant pathways that would result in great losses of time and money. When intending to assess an ATMP in a clinical trial, the IMPD is the most complex and important document to develop. Its structure provides researchers with guidance regarding the preclinical data needed to generate a safe and effective IMP. Key steps along the process include scalability and GMP manufacturing, along with animal models to perform PD, PK, and Tox studies.

## Author Contributions

PG and CG-M conceived the presented idea. PG wrote the manuscript with support from CG-M, CP-V, JV, and RC. CG-M, JV, and AB-G performed a critical revision of the manuscript. All authors gave final approval of the version to be published.

## Conflict of Interest

The authors declare that the research was conducted in the absence of any commercial or financial relationships that could be construed as a potential conflict of interest.

## Abbreviations

AEMPS: Spanish Agency of Medicines and Medical Devices
AEMPS: Agencia Española del Medicamento y Producto Sanitario
ATMP: Advanced Therapy Medicinal Product
MTA: Medicamento de Terapia Avanzada
CTD: Common Technical Document
IMP: Investigational Medicinal Product
PEI: Producto En fase de Investigación
IMPD: Investigational Medicinal Product Dossier
EPEI: Expediente de Producto En fase de Investigación
TPP: Target Product Profile
FDP: Ficha de Desarrollo del Producto.
